# Loss of skeletal muscle index and survival in patients with metastatic colorectal cancer: Secondary analysis of the phase 3 CAIRO3 trial

**DOI:** 10.1002/cam4.2787

**Published:** 2019-12-18

**Authors:** Sophie A. Kurk, Petra H. M. Peeters, Bram Dorresteijn, Pim A. de Jong, Marion Jourdan, Geert‐Jan M. Creemers, Frans L. G. Erdkamp, Felix E. de Jongh, Peter A. M. Kint, Boelo J. Poppema, Sandra A. Radema, Lieke H. J. Simkens, Bea C. Tanis, Manuel L. R. Tjin‐A‐Ton, Ankie Van Der Velden, Cornelis J. A. Punt, Miriam Koopman, Anne M. May

**Affiliations:** ^1^ Department of Medical Oncology University Medical Center Utrecht Utrecht University Utrecht The Netherlands; ^2^ Department of Epidemiology Julius Center for Health Sciences and Primary Care Utrecht University Utrecht The Netherlands; ^3^ Danone Nutricia Research Nutricia Advanced Medical Nutrition Utrecht The Netherlands; ^4^ Department of Radiology University Medical Center Utrecht Utrecht University Utrecht The Netherlands; ^5^ Department of Medical Oncology Catharina Hospital Eindhoven The Netherlands; ^6^ Department of Medical Oncology Zuyderland Hospital Sittard The Netherlands; ^7^ Department of Medical Oncology Ikazia Hospital Rotterdam The Netherlands; ^8^ Department of Radiology Amphia Hospital Breda The Netherlands; ^9^ Department of Radiology Department of Medical Oncology Ommelander Hospital Group Groningen The Netherlands; ^10^ Department of Medical Oncology Radboud University Medical Center Nijmegen The Netherlands; ^11^ Department of Medical Oncology Maxima Medical Center Eindhoven The Netherlands; ^12^ Department of Medical Oncology Groene Hart Hospital Gouda The Netherlands; ^13^ Department of Medical Oncology Rivierenland Hospital Tiel the Netherlands; ^14^ Department of Medical Oncology Tergooi Hospital Blaricum The Netherlands; ^15^ Department of Medical Oncology Amsterdam University Medical Center University Amsterdam Amsterdam The Netherlands

**Keywords:** Body composition, body mass index, chemotherapy, metastatic colorectal cancer, sarcopenia, skeletal muscle mass, survival

## Abstract

**Background:**

Low skeletal muscle index (SMI) in metastatic colorectal cancer (mCRC) patients is associated with poor outcomes. The prognostic impact of SMI changes during consecutive palliative systemic treatments is unknown.

**Methods:**

This is a retrospective analysis of the phase 3 CAIRO3 study. The CAIRO3 study randomized 557 patients between maintenance capecitabine + bevacizumab (CAP‐B) or observation, after six cycles capecitabine + oxaliplatin + bevacizumab (CAPOX‐B). Upon first disease progression (PD1), CAPOX‐B was reintroduced until second progression (PD2). SMI was assessed by computed tomography (CT) (total 1355 scans). SMI and body mass index (BMI) changes were analyzed for three time‐periods; p1: during initial CAPOX‐B, p2: randomization to PD1, and p3: PD1 to PD2. The association between absolute and change in SMI and BMI (both per 1 standard deviation) during p1‐p3, with PD1, PD2, and survival was studied by Cox regression models.

**Results:**

This analysis included 450 of the 557 patients randomized in the CAIRO3 study. Mean SMI decreased during p1: mean −0.6 SMI units [95% CI −1.07;‐0.26] and p3: −2.2 units [−2.7;‐1.8], whereas during p2, SMI increased + 1.2 units [0.8‐1.6]. BMI changes did not reflect changes in SMI. SMI loss during p2 and p3 was significantly associated with shorter survival (HR 1.19 [1.09‐1.35]; 1.54 [1.31‐1.79], respectively). Sarcopenia at PD1 was significantly associated with early PD2 (HR 1.40 [1.10‐1.70]). BMI loss independent of SMI loss was only associated with shorter overall survival during p3 (HR 1.35 [1.14‐1.63]).

**Conclusions:**

In mCRC patients, SMI loss during palliative systemic treatment was related with early disease progression and reduced survival. BMI did not reflect changes in SMI and could not identify patients at risk of poor outcome during early treatment lines.

## 1 INTRODUCTION

In metastatic colorectal cancer (mCRC) patients, the identification of predictive and potentially modifiable characteristics that predispose to poor treatment outcomes is important to improve outcomes, quality of care, and reduce health‐care costs.^1^

An interesting novel prognosticator is the loss of skeletal muscle mass, as muscle mass loss in cancer (including mCRC) patients can be easily evaluated using computed tomography (CT) scans.^2^, ^3^ During advanced cancer, muscle mass loss is the result of a multifactorial syndrome in which a reduced nutritional intake, metabolic changes (due to tumor and oncologic treatment), often combined with low physical activity levels, lead to alterations in body composition and eventually to cancer cachexia.^4^ Loss of muscle mass, alone or as a part of cancer cachexia, is easily overlooked in the presence of obesity, negatively impacts patients' prognosis and quality of life, and potentially amendable to treatment.^4^, ^5^, ^6^, ^7^, ^8^ In several studies including mCRC patients, low skeletal muscle mass (sarcopenia) was associated with poor survival.^6^, ^9^, ^10^ Furthermore, a decrease in muscle mass over time was associated with poor survival.^7^, ^11^, ^12^, ^13^ Interestingly, these studies only investigated muscle mass changes between two time points and its association with disease outcome. No studies have investigated the evolution of muscle mass on multiple time points, during consecutive treatment regimens, and how, within each regimen, these changes relate to (progression free) survival. This is of particular relevance shortly after the mCRC diagnosis, when an anabolic response is more likely to occur and thus change in muscle mass is potentially modifiable by interventions that aim to improve muscle mass.^7^, ^14^

Recently, we observed that patients significantly lost muscle mass during six cycles of first‐line capecitabine + oxaliplatin+bevacizumab (CAPOX‐B), despite a good response to treatment.^15^ Interestingly, during subsequent less intensive maintenance capecitabine + bevacizumab (CAP‐B) or observation, muscle mass recovered to the levels at the start of initial CAPOX‐B. Finally, after reintroduction of more intensive CAPOX‐B or other treatment, patients again lost muscle mass. Here, we investigate how these longitudinal muscle changes during the consecutive treatments relate to outcome, in particular, to time to disease progression and overall survival. Furthermore, in advanced cancer patients, including CAIRO3 patients, body weight changes over time may not correlate with muscle mass changes.^11^, ^14^ Also, muscle mass and fat mass have been found to respond differently to tumor and treatment, and are thus differently associated with cancer patients' outcome.^3^, ^14^, ^16^ Therefore, as a secondary aim, we investigated longitudinal body mass index (BMI) changes, including BMI changes adjusted for muscle mass changes (ie, as a surrogate for fat mass), to study their relation with survival outcomes.

## 2 METHODS

### 2.1 Patients

**Figure 1:**
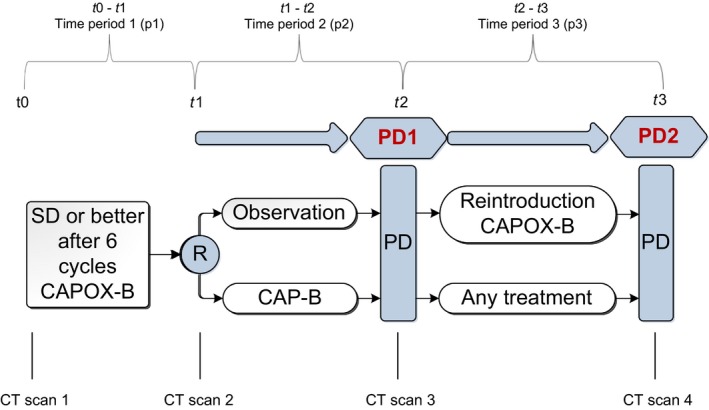
Design CAIRO3 study. CAIRO3 study design and time points in which CT scans were collected. At these time points also BMI measures were collected

Patients participated in the randomized phase 3 CAIRO3 study,^17^ conducted by the Dutch Colorectal Cancer Group (DCCG). CAIRO3 investigated the effect of maintenance CAP‐B vs observation in mCRC patients with stable disease (or better) after first‐line six cycles CAPOX‐B (Figure 1). Main eligibility criteria were ≥18 years, histologically confirmed CRC, WHO performance status ≤1, and no previous mCRC treatment. After randomization, disease status was assessed every 9 weeks using CT scans and the Response Evaluation Criteria In Solid Tumors (RECIST) version 1.1,^18^ or when disease progression was suspected due to clinical symptoms. Upon first disease progression (PD1), patients received CAPOX‐B or other reintroduction treatment until the second disease progression (PD2). The CAIRO3 protocol was approved by the Medical Ethics Committee of Nijmegen, The Netherlands and registered at ClinicalTrials.gov, number http://clinicaltrials.gov/show/NCT00442637.

### 2.2 Skeletal muscle measurements

CT scans were collected at four time points: before start of initial six cycles of CAPOX‐B (t0), at randomization between CAP‐B and observation (t1), at PD1, before start of CAPOX‐B or other reintroduction treatment (t2), and at PD2, after progression on any systemic reintroduction treatment (t3) (Figure 1). Thereby three treatment periods were defined: initial six cycles of CAPOX‐B (p1), maintenance CAP‐B or observation (p2), and CAPOX‐B or other reintroduction treatment (p3).

Skeletal muscle mass measurements were performed by a trained analyst and the Slice‐o‐matic software (Tomovision). Detailed information on the muscle mass measurements is described elsewhere.^15^ In short, the surface area of muscle compartments was quantified from single slices at the third vertebral level (L3) using thresholds in Hounsfield units (HU) (−29 to 150 HU).^3^, ^19^, ^20^ The skeletal muscle index (SMI) was defined as the L3 muscle area (cm^2^) divided by patient height (m^2^).^3^, ^9^, ^19^

### 2.3 Other anthropometric measures

Height and body weight at time points t0‐t4 were retrieved from medical records to calculate BMI.^21^ Sarcopenia status was determined by published sex‐specific cutoff points.^9^ Sarcopenic obesity^22^ was defined by being sarcopenic and a BMI ≥ 30.

### 2.4 Statistical analysis

Detailed changes in skeletal muscle mass and body weight during CAIRO3 treatments were previously reported.^15^ We additionally analyzed changes in SMI and BMI during these treatments by linear mixed effects models.^23^ The baseline value of the outcome (SMI/BMI) was included in the outcome vector. Potential confounders were age, sex, lactate dehydrogenase (LDH) levels, best response to initial six cycles CAPOX‐B, resection of the primary tumor, and the number of metastatic sites. The final model was selected based on the Akaike Information Criterion (AIC) and included: treatment arm, time, age, sex, resection primary tumor, and the interaction of treatment arm by time as fixed effects. Modeling time as a random effect did not increase the model fit as indicated by the AIC. To investigate differences in SMI and BMI changes over time between the CAIRO3 treatment arms, we checked the significance of the two‐way interaction including treatment arm by time.

Next, we examined the association of SMI and BMI (absolute, categories, sarcopenia yes/no, or loss continuously per 1 standard deviation (SD)) with time to progression and death, using multivariable Cox proportional hazard regression models. All models were adjusted for predefined prognostic factors: age (continuously), sex, WHO performance status (0/1), stage (1‐4), primary tumor site (colon/sigmoid/rectum), resection primary tumor (yes/no), response to initial treatment (stable disease/partial or complete response), LDH level at randomization (normal/elevated), synchronous vs metachronous mCRC, and dose reductions during initial treatment (yes/no). Changes in SMI and BMI were additionally adjusted for the time within which the changes occurred. In additional analyses, the models including BMI loss were adjusted for SMI loss, to investigate the relation of BMI loss independent of SMI loss, with outcomes. We used separate models for each treatment interval, setting the time at 0 at each interval start, and calculated hazard ratios with corresponding 95% confidence intervals for the associations of absolute SMI and BMI (at t0, t1, t2) or change in SMI and BMI (during p1, p2, and p3) with PD1, PD2, and death. Due to the low number of patients with underweight or sarcopenic obesity on the different time points, these patients were excluded from the corresponding analysis.^15^

To investigate whether the associations of BMI or SMI loss with survival were different between patients randomized to the different CAIRO3 arms, we checked the significance of the two‐way interactions including BMI, SMI loss, or sarcopenia by arm. Finally, to check whether results differed between patients with available BMI measures and with available SMI measures, we repeated the previous analyses, excluding patients with missing SMI or BMI measures. All p‐values were two‐sided, and the level of significance was considered at *P* < .05. SPSS version 21 was used.

## 3 RESULTS

### 3.1 Patients

**Figure 2:**
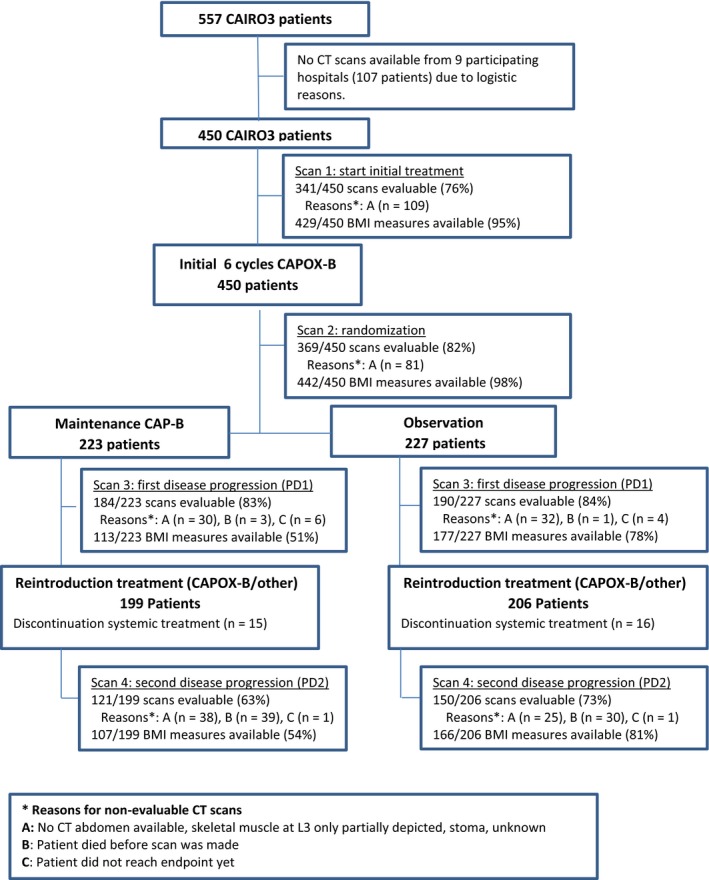
Flowchart. Evaluable CT scans per time point during CAIRO3, including reasons for nonevaluable CT scans

Routine CT scans were available from 450 of 557 (81%) CAIRO3 patients. In total, 1355 CT scans were available for skeletal muscle analysis and 1434 body weight measures were available for BMI analysis (Figure 2).

Table 1 describes the baseline patient and treatment characteristics. Patients were on average 64 ± 9 years of age and 63% were male. The mean BMI was 26.0 ± 4.3 kg/m^2^ and 51% were sarcopenic. Six percent had sarcopenic obesity. Sarcopenic compared to non‐sarcopenic patients had a lower mean BMI (25.9 vs 27.2 kg/m^2^) and were less frequently obese (8% vs 20%). The percentage of underweight patients was comparable (4% vs 1%).

**Table 1:** Baseline patient and treatment characteristics

	Total group	By treatment arm		By sarcopenia status	
	Total group (n = 450)	Maintenance CAP‐B (n = 223)	Observation arm (n = 227)	Non‐sarcopenic (n = 162)	Sarcopenic (n = 171)
Age, mean in years (±SD)	64 (±9)	63 (±9)	64 (±9)	62 (±9)	64 (±9)
≤70	332 (74%)	171 (77%)	161 (71%)	127 (78%)	119 (70%)
>70	118 (26%)	52 (23%)	66 (29%)	35 (22%)	52 (30%)
Sex					
Female	165 (37%)	82 (37%)	83 (37%)	55 (34%)	70 (41%)
Male	285 (63%)	141 (63%)	144 (63%)	107 (66%)	101 (59%)
Primary site					
Colon only	226 (50%)	109 (49%)	117 (52%)	78 (48%)	92 (54%)
Rectum only	131 (79%)	69 (31%)	62 (27%)	47 (29%)	45 (26%)
Rectosigmoid	93 (21%)	45 (20%)	48 (21%)	37 (23%)	34% (21%)
Resection primary tumor					
Yes	270 (60%)	131 (59%)	139 (61%)	60 (37%)	78 (46%)
No	180 (40%)	92 (41%)	88 (39%)	102 (63%)	93 (54%)
WHO performance score					
0	281 (62%)	139 (62%)	142 (63%)	101 (62%)	102 (60%)
1	169 (38%)	84 (38%)	85 (37%)	61 (38%)	69 (40%)
Number of metastatic sites					
1	196 (45%)	102 (47%)	94 (44%)	77 (49%)	71 (44%)
>1	235 (55%)	113 (53%)	122 (56%)	79 (51%)	91 (56%)
Unknown	19	8	11	6	9
LDH (IU/L)					
Elevated	252 (56%)	125 (56%)	127 (56%)	72 (44%)	81 (47%)
Normal	198 (44%)	98 (44%)	100 (44%)	90 (56%)	90 (53%)
BMI, kg/m^2^ a					
Underweight (<18.5)	10 (2%)	5 (2%)	5 (2%)	1 (1%)	6 (4%)
Normal (18.5‐25)	185 (43%)	86 (40%)	99 (45%)	69 (43%)	74 (45%)
Overweight (25‐30)	166 (38%)	79 (37%)	87 (40%)	58 (37%)	72 (43%)
Obese (30+)	68 (16%)	40 (19%)	28 (13%)	31 (20%)	14 (8%)
Unknown	21	13	8	3	5
Sarcopenia^a^, ^b^					
Yes	171 (51%)	89 (53%)	82 (50%)		
No	162 (49%)	80 (46%)	82 (50%)	NA	NA
Unknown	117	54	63		
Sarcopenic obesity^a^, ^c^					
Yes	14 (4%)	9 (6%)	5 (3%)		14 (8%)
No	311 (96%)	156 (95%)	155 (97%)	NA	152 (92%)
Unknown	125^d^	58^d^	67^d^		5^d^
Best response to initial CAPOX‐B treatment					
Complete response/ partial response	298 (66%)	150 (67%)	148 (65%)	102 (63%)	118 (69%)
Stable disease	152 (34%)	73 (33%)	79 (35%)	60 (37%)	53 (31%)
CAIRO3 arm					
Observation	227 (50%)	NA	NA	82 (51%)	82 (48%)
Cap‐B	223 (50%)			80 (49%)	89 (52%)
Reintroduction treatment					
CAPOX‐B	255 (57%)	118 (46%)	137 (54%)	72 (44%)	79 (46%)
Other	195 (43%)	105 (54%)	90 (46%)	90 (56%)	92 (54%)

Note

All measurements were collected at time of randomization, unless otherwise specified.

Abbreviations: BMI, body mass index; CAP‐B, capecitabine + bevacizumab; CAPOX‐B, capecitabine + oxaliplatin+bevacizumab; WHO, World Health Organization, SD, standard deviation.

a Determined at start of initial treatment with six cycles CAPOX‐B

b Sarcopenia was defined as skeletal muscle index (SMI) of <43 cm^2^/m^2^ for males with body mass index (BMI) <25 cm^2^/m^2^, <53 cm^2^/m^2^ for males with body mass index (BMI) ≥25 cm^2^/m^2^, and < 41 cm^2^/m^2^ for females.^9^

c Sarcopenic obesity was defined as being sarcopenic and BMI > 30 cm^2^/m^2^.

d Higher number of missings in sarcopenic obesity status compared to “normal” sarcopenia status, because of the higher number of missings of the BMI values in females. In female patients, BMI is necessary to determine sarcopenic obesity status, but not to determine sarcopenia status.

### 3.2 SMI and BMI changes during treatments

**Figure 3:**
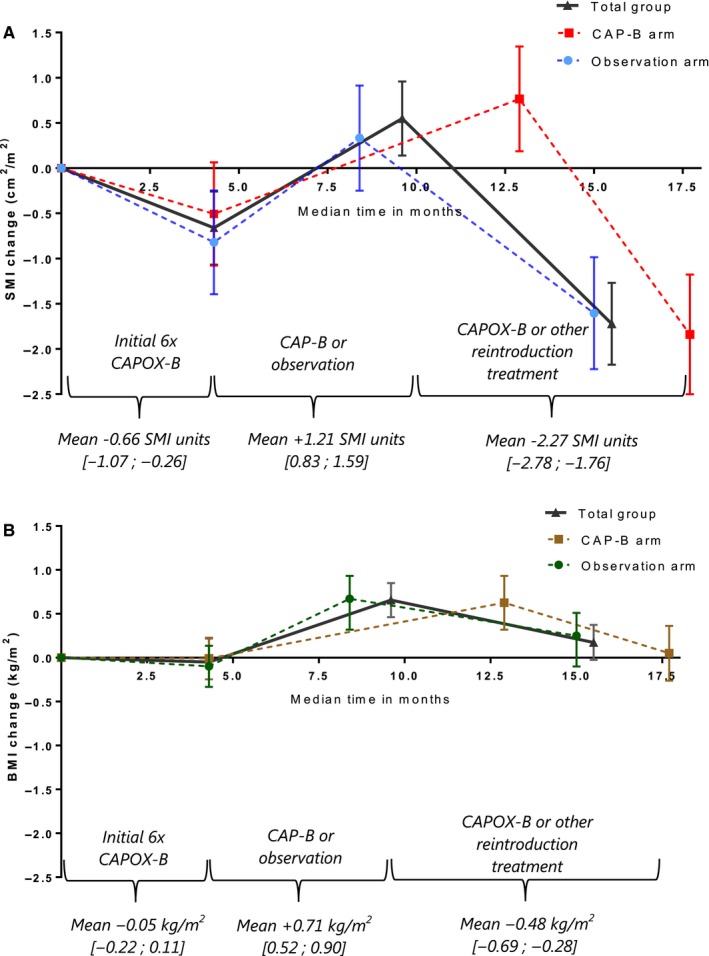
SMI and BMI changes during palliative systemic treatment. A, This figure displays the modeled mean skeletal muscle index (SMI) changes with 95% confidence interval bars during palliative systemic, for the total group and per treatment arm. B, This figure displays the modeled mean body mass index (BMI) changes with 95% confidence interval bars during palliative systemic treatment, for the total group and per treatment arm. Abbreviations: CAPOX‐B, capecitabine + oxaliplatin+bevacizumab; CAP‐B, capecitabine + bevacizumab

We observed significant SMI and BMI changes during p1, p2, and p3 (Figure 3).^15^ During p1 (initial six cycles of CAPOX‐B), mean SMI decreased (−0.66 [−1.07;‐0.26]), whereas BMI remained stable (−0.05 [−0.22; 0.11]). During p2 (CAP‐B or observation), SMI and BMI increased (+1.21 [0.82;1.60] and + 0.71 [0.52‐0.90], respectively). During p3 (CAPOX‐B/ other treatment), SMI and BMI decreased (−2.25 [−2.68; −1.81] and −0.48 [−0.69;‐0.28], respectively). Mean BMI and SMI changes over time did not differ significantly between the randomized treatment arms (SMI p_interaction_ = 0.80; BMI p_interaction_ = 0.58).

### 3.3 Association with disease progression and overall survival

For all patients, median times from start of initial treatment (t0) to randomization (t1), PD1 (t2), PD2 (t3), and death were 4.3 months (Q1‐Q3 4.0‐4.5), 9.6 months (6.8‐15.0), 17.0 months (12.0‐23.7), and 24.6 months (16.1‐35.7), respectively.

During p1 (initial six cycles CAPOX‐B), absolute SMI and BMI at start (t0), and SMI loss and BMI loss during treatment were not associated with progression or death (Table 2).

**Table 2:** Associations of absolute and change in SMI and BMI with disease progression and death

	Patients at risk (n)	HR first disease progression (95% CI)	HR second disease progression (95% CI)	HR death (95% CI)
At start of six cycles initial CAPOX‐B				
SMI continuous per SD (8.5) lower	341	1.02 (0.88‐1.14)	1.01 (0.86‐1.13)	1.03 (0.90‐1.18)
Sarcopenia^a^	171	1.01 (0.86‐1.37)	1.16 (0.92‐1.45)	1.07 (0.84‐1.35)
Non‐sarcopenia^a^	162	[reference]	[reference]	[reference]
BMI continuous per SD (4.3) lower	429	1.00 (0.89‐1.11)	0.96 (0.89‐1.12)	0.96 (0.89‐1.13)
Normal weight	185	[reference]	[reference]	[reference]
Overweight	166	0.94 (0.75‐1.18)	0.84 (0.68‐1.07)	0.97 (0.77‐1.21)
Obese	68	0.95 (0.69‐1.29)	0.94 (0.69‐1.28)	1.13 (0.83‐1.53)
Change during initial CAPOX‐B				
SMI loss per SD (3.7)	300	1.01 (0.88‐1.15)	0.94 (0.82‐1.08)	0.91 (0.78‐1.04)
BMI loss per SD (1.6)	421	1.02 (0.91‐1.15)	1.01 (0.90‐1.13)	0.94 (0.84‐1.05)
BMI loss per SD adjusted for SMI loss per SD	282	1.09 (0.93‐1.28)	1.13 (0.96‐1.32)	0.97 (0.83‐1.13)
Start maintenance CAP‐B/observation				
SMI continuous per SD (8.0) lower	369	1.02 (0.86‐1.12)	1.03 (0.90‐1.18)	1.02 (0.88‐1.15)
Sarcopenia^a^	203	1.02 (0.82‐1.27)	1.06 (0.85‐1.32)	1.01 (0.87‐1.36)
Non‐sarcopenia^a^	164	[reference]	[reference]	[reference]
BMI continuous per SD (4.2) lower	442	NA	1.00 (0.90‐1.12)	0.99 (0.88‐1.11)
Normal weight	189	[reference]	[reference]	[reference]
Overweight	160	1.08 (0.86‐1.36)	1.05 (0.84‐1.32)	1.10 (0.88‐1.38)
Obese	65	1.07 (0.79‐1.45)	0.98 (0.72‐1.33)	1.10 (0.81‐1.49)
Change during maintenance CAP‐B and observation				
SMI loss per SD (3.2)	322	NA	1.09 (0.95‐1.23)	**1.19 (1.09‐1.35)**
BMI loss per SD (1.7)	283	NA	**1.15 (1.01‐1.33)**	1.09 (0.94‐1.25)
BMI loss per SD adjusted for SMI loss per SD	215	NA	1.18 (0.97‐1.41)	0.99 (0.83‐1.18)
Start reintroduction of CAPOX‐B or other treatment				
SMI continuous per SD (8.5) lower	374	NA	**1.16 (1.02‐1.32)**	1.14 (0.99‐1.30)
Sarcopenia^a^	143	NA	**1.40 (1.10‐1.70)**	1.20 (0.96‐1.54)
Non‐sarcopenia^a^	184	[reference]	[reference]	[reference]
BMI continuous per SD (4.0) lower	290	NA	0.97 (0.85‐1.10)	0.94 (0.83‐1.08)
Normal weight	89	NA	[reference]	[reference]
Overweight	143	NA	0.90 (0.68‐1.17)	0.98 (0.74‐1.29)
Obese	44	NA	0.95 (0.66‐1.36)	0.98 (0.67‐1.44)
Change during CAPOX‐B or other reintroduction treatment				
SMI loss per SD (3.1)	246	NA	NA	**1.54 (1.31‐1.79)**
BMI loss per SD (1.5)	273	NA	NA	**1.25 (1.09‐1.43)**
BMI loss per SD adjusted for SMI loss per SD	191	NA	NA	**1.35 (1.14‐1.63)**

Note

HR adjusted for: time within which the muscle changes occurred, age, sex, WHO performance status, stage, primary tumor site, resection primary tumor, response to initial treatment, LDH at randomization, synchronous vs metachronous metastatic colorectal cancer, dose reduction during the initial six cycles CAPOX‐B treatment. In bold, statistically significant HR.

Abbreviations: 95% CI, 95% confidence interval; HR, hazard ratio; IQR, interquartile range; NA, not applicable; SD, standard deviation; SMI, Skeletal muscle index.

a Sarcopenia was defined as skeletal muscle index (SMI) of <43 cm^2^/m^2^ for males with body mass index (BMI) <25 cm^2^/m^2^, <53 cm^2^/m^2^ for males with body mass index (BMI) ≥25 cm^2^/m^2^, and <41 cm^2^/m^2^ for females.^9^

During p2 (maintenance CAP‐B/observation), again absolute SMI and BMI at the start (t1) of treatment were not associated with early progression and death. In contrast, SMI loss and BMI loss during treatment were associated with early progression and death. Significant associations were found between SMI loss per SD and shorter survival (HR 1.19 [1.09‐1.35]) and between BMI loss per SD and early PD2 (HR 1.15 [1.01‐1.33]). The association between BMI loss per SD and early PD2 disappeared after adjusting for SMI loss (HR 1.18 [0.97‐1.41]).

During p3 (reintroduction treatment), absolute SMI, including sarcopenia, but not BMI, at start (t2) was significantly associated with early PD2 (SMI HR 1.16 [1.02‐1.32] and sarcopenia HR 1.40 [1.10‐1.70]) and nonsignificantly associated with death (SMI HR 1.14 [0.99‐1.30] and sarcopenia HR 1.20 [0.96‐1.54]). Change in SMI and BMI (±adjusted for SMI loss) was significantly associated with early death (HR per SD loss in SMI 1.54 [1.31‐1.79], BMI 1.25 [1.09‐1.43], and BMI adjusted for SMI 1.35 [1.14‐1.63]).

Results did not differ by CAIRO3 treatment arm (all p_interaction_ > 0.05). Finally, the analyses including only those patients who had both SMI and BMI measures showed comparable results (data not shown).

## 4 DISCUSSION

In this large longitudinal study in mCRC patients, we provide a unique insight into SMI and BMI changes during various palliative systemic treatments, starting from first‐line treatment, and show how these changes relate to time to progression and survival. Absolute SMI (including sarcopenia), and SMI loss during subsequent treatments, including observation, related to shorter time to progression and shorter overall survival. In contrast, absolute BMI or BMI loss independent of SMI loss was not associated with early progression or death, except for BMI loss independent of SMI loss during CAPOX‐B reintroduction treatment and overall survival.

Several previous studies have described a relationship between muscle mass loss in mCRC patients and poor survival.^7^, ^11^, ^12^, ^13^ Most studies included heterogeneous patient populations receiving different treatments and determined muscle mass at different time points during the disease. However, muscle mass loss depends on tumor type and oncologic treatment.^8^, ^15^, ^24^ Furthermore, muscle mass loss is mostly observed during periods of disease progression^7^, ^12^, ^14^ and most studies consisted of a proportion of patients with (very) advanced metastatic cancer. Hence, the inclusion of patients with short life expectancy may be in part responsible for the observed association between muscle mass loss and survival in these studies. In contrast, we analyzed a large and homogeneous population of mCRC patients from a prospective randomized phase 3 study with standardized assessment of disease progression. Furthermore, patients received defined consecutive systemic treatments starting from first‐line treatment, which help to reduce potential confounding by treatment. For example when patients with poor prognosis (and possibly more SMI loss) receive different treatments compared to patients with a better prognosis (and possibly less muscle loss). We found that SMI loss during first‐line (maintenance/observation) and reintroduction treatment (CAPOX‐B or other) was associated with shorter overall survival. Moreover, absolute SMI at PD1 was associated with early PD2. Hence, SMI loss carries important prognostic information during the initial phase of mCRC, and thus also applies to mCRC patients who responded to initial CAPOX‐B and therefore have a relatively favorable prognosis. Even more, SMI loss varied over time and per regimen. This further supports the hypothesis that muscle mass during mCRC can be modified and, thereby, can potentially improve outcome.

During advanced cancer, involuntary weight loss is considered an important diagnostic feature of cancer cachexia, and thereby the most frequently (and sometimes only) used clinical parameter to identify patients at risk of poor outcome due to their nutritional status.^4^, ^5^ However, when weight loss becomes prominent, the window of opportunity for rehabilitation of patients may have been passed.^4^ Our data show that throughout CAIRO3 follow‐up, overall BMI changes did not reflect the ongoing SMI changes over time. This is in line with previous studies that found that during cancer progression, patients do not necessarily lose or gain fat and muscle mass in equal proportions when their BMI changes.^3^, ^14^, ^16^ Indeed, fat can be gained while muscle is being lost^7^ and body weight is, therefore, not very informative on muscle mass changes. Furthermore, in contrast to early SMI change, early BMI changes did not relate to poor outcome. Hence, SMI measures, which can be easily assessed using routine CT scans, provide important additional information beyond BMI, and have the potential of identifying patients at risk of poor disease outcome, already during early stage mCRC.^2^

During CAIRO3, SMI loss was more likely to occur during high intensive treatments (CAPOX‐B). During subsequent low intensive CAP‐B or observation, SMI loss was on average reversible. This implies that during early mCRC, specifically, therapeutic effects may be important contributors to SMI loss. Different causes of muscle loss may benefit from different treatments and may have different prognosis.^4^, ^14^ However, separating muscle loss due to treatment effects from other causes is difficult. Even more, the stepwise decline in muscle mass loss that occurs when patients receive subsequent anticancer therapies may lead to sarcopenia and conversely poor outcomes. Therefore, treatment‐associated muscle wasting should be considered important during nutritional status assessment.^25^

In contrast to results of previous studies in mCRC patients,^26^, ^27^ we did not observe an association between sarcopenia and reduced survival, or SMI loss during initial six cycles CAPOX‐B and reduced survival. A possible explanation is that the CAIRO3 study consists of patients with a relatively good prognosis, since patients who showed disease progression or experienced inacceptable toxicity during initial CAPOX‐B were excluded.

We observed no differences in the association of SMI loss and survival between patients randomized to maintenance treatment or observation. Maintenance treatment consisted of bevacizumab plus a continuous low dose of capecitabine, and seems to have no impact on SMI. During both CAP‐B or observation, patients on average had the capability to recover in SMI to their baseline (t0) SMI levels. However, when SMI loss was observed in patients irrespective of treatment or observation, this was significantly associated with poor survival.

Exact mechanisms which link SMI loss to shorter survival are unknown. Two mechanisms, cancer cachexia and altered drug pharmacokinetics, may contribute to the reduced survival in cancer patients.^5^, ^14^ Muscle mass loss is a hallmark of cancer cachexia affecting 50%‐80% of mCRC patients.^5^, ^14^ Cachexia is associated with negative clinical consequences including physical impairment, poor quality of life, reduced treatment tolerance, and eventually shorter survival.^14^ Furthermore, low muscle mass is hypothesized to alter pharmacokinetic parameters such as the volume of distribution and increased drug exposure.^22^ This could lead to increased treatment‐related toxicities and thereby more dose adaptations.^22^, ^28^, ^29^, ^30^ Eventually, this can result in a compromised treatment efficacy and ultimately reduced survival.

To counteract muscle loss, several therapeutic approaches can be considered, including physical exercise, nutritional supplements (high‐energy enriched with high‐protein, *n*‐3 PUFA‐enriched), and orexigenic agents.^5^, ^8^ A matter of debate is whether preventing muscle mass loss during cancer(treatment) also improves prognosis, since studies on this topic are limited. A few studies on nutritional support did report improvement in some aspects of quality of life,^8^ and one study reported a benefit on disease‐free survival in mCRC.^31^ Furthermore, physical exercise interventions and orexigenic agents were described to increase muscle mass and muscle strength in various cancer types, but the effect on clinical outcome was not evaluated.^32^, ^33^ Previous studies were not designed and thus powered for the evaluation of the effect of these interventions on clinical outcomes such as disease progression and overall survival, and had large heterogeneity in the study population. Furthermore, most clinical trials (excluding exercise trials) have been conducted in patients with quite advanced disease who, therefore, may have been less responsive to interventions.^5^ Finally, due to the multifactorial origin of muscle loss in cancer patients, better (if not any) results are expected when combining different procedures in a multimodal approach.^5^, ^8^, ^14^

We are aware of several limitations of this study. Firstly, the observational nature of our study does not allow us to draw conclusions on causal relationships. Unmeasured confounding and/or reversed causality (when a more aggressive cancer leads to lower SMI) may have contributed to the observed associations.^34^ Secondly, we did not perform fat tissue measurements, but used BMI measures adjusted for SMI as a surrogate for fat tissue instead. The reason for this is that during the CT scan analyses, we observed that organ positioning (eg, bowel extension) impacted on repeated single‐slice fat measurements, and therefore deemed as not reliable. The disadvantage of using BMI and not fat tissue measurements is that we cannot control for other causes of weight gain, including edema and ascites. Thirdly, we included a large number of 450 patients in our analysis and for most patients repeated BMI and SMI measures were available. We performed multiple analyses on the relation of BMI and SMI (changes during treatment) and their relation with outcome, all showing the same direction of the associations. We expect that including more patients would probably lead to more precision and not to different results. Finally, we excluded patients for whom a CT scan was not available due to death or discontinuation of systemic treatment at PD1 and PD2, which may have led to an underestimation of our association (ie, when excluded patients without CT scan were also the patients with poor prognosis). However, baseline characteristics of patients with available CT scans were comparable to patients without available CT scans and we adjusted for potential confounders.

In conclusion, in mCRC patients with good response to initial six cycles of CAPOX‐B treatment, SMI loss during palliative systemic treatment varies per regimen. We show that a relative simple measure, such as single‐slice body composition analysis using routine CT scans, can detect sometimes otherwise occult SMI loss. The reported SMI loss was related to shorter overall survival during first‐line maintenance CAP‐B or observation and during CAPOX‐B or other reintroduction treatment, whereas sarcopenia at start of CAPOX‐B or other reintroduction treatment was associated with early disease progression. BMI did not accurately reflect changes in SMI and did not identify patients at risk of poor outcome during the early mCRC treatment periods. The main next question is whether targeting muscle mass loss (eg, with exercise and nutritional interventions) in mCRC patients could be a valuable intervention to improve treatment outcomes. To this end, prospective well‐powered intervention studies are needed to investigate the effect of SMI preservation on outcome, including disease progression and overall survival.

## DISCLAIMERS

Bram Dorresteijn and Marion Jourdan work at Nutricia Research.
